# *Paliurus* Fruits from the Oligocene of South China and Their Phytogeographic Implications

**DOI:** 10.1371/journal.pone.0140653

**Published:** 2015-11-04

**Authors:** Jun-Ling Dong, Bai-Nian Sun, Fu-Jun Ma, Qiu-Jun Wang, Pei-Hong Jin, Wen-Jia Wang, Peng Deng, Yi Yang, Xiao-Jing Li

**Affiliations:** 1 School of Earth Sciences & Key Laboratory of Mineral Resources in Western China (Gansu Province), Lanzhou University, Lanzhou, 730000, China; 2 Key Laboratory of Economic Stratigraphy and Palaeogeography, Nanjing Institute of Geology and Palaeontology, CAS, Nanjing, 210008, China; Institute of Botany, CHINA

## Abstract

*Paliurus favonii* Unger is recognized and described based on fruits from the Oligocene Ningming flora of Guangxi, South China. Characteristics of the present specimens include circular winged fruits that are 10.0–11.5 mm in diameter with a central endocarp at 3.0 to 4.0 mm in diameter. The specimens fall into the morphological range of the fossil species *P*. *favonii*, which has been observed in other Cenozoic sites in the Northern Hemisphere. The present discovery represents the lowest latitude distribution of *P*. *favonii* in the world, and we are presenting the first *P*. *favonii* fossil described with detailed cuticular characteristics from China. Further, this finding demonstrates that the genus existed in the Oligocene Ningming region, South China, and provides new information for understanding the fossil history. The dispersal mode for winged fossils demonstrates that wind dispersal is well-represented in the Oligocene Ningming flora.

## Introduction

**Fig 1:**
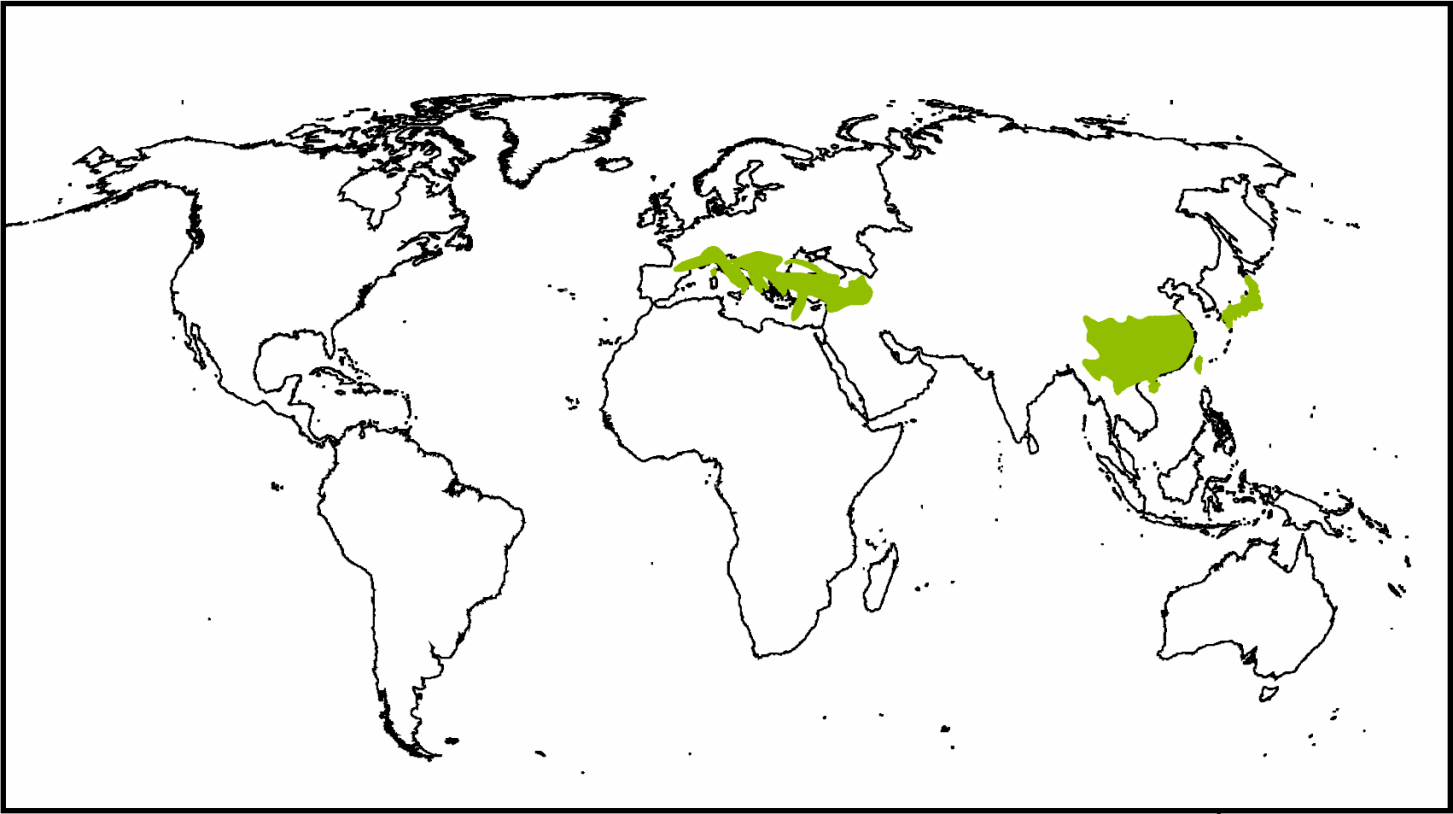
Geographic distribution of extant *Paliurus* [1, 4].

**Fig 2:**
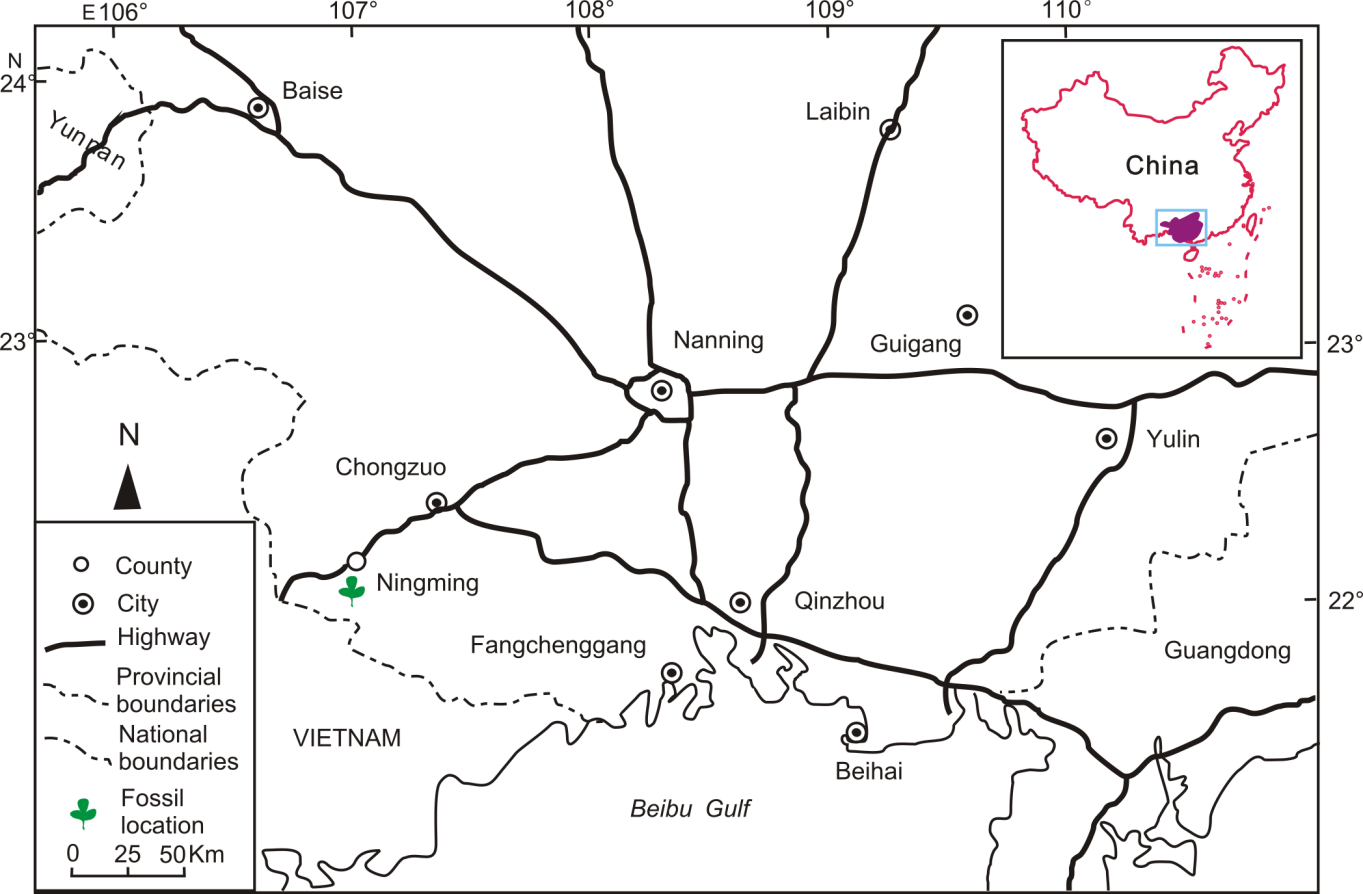
Fossil locality showing Ningming County of Guangxi, South China.

*Paliurus* Mill. of the plant family Rhamnaceae contains five extant species of deciduous or evergreen shrubs and trees. The genus is distributed in southern portions of Europe (Northern and Eastern Mediterranean, as well as parts of Northern Africa and eastward to Western Asia) and Asia (China, Korea, Japan, and Vietnam; Fig 1; [1, 2]), and most occurrences are limited to a moist, equable, and temperate climate in tropical to warm temperate regions [3, 4]. *P*. *hirsutus* Hemsl., *P*. *orientalis* (Franch.) Hemsl. and *P*. *hemsleyanu*s Rehder ex. Schirarend & Olabi are endemic to China, while *P*. *ramosissimus* (Lour.) Poir. occurs in southern China, Korea, Japan and Vietnam. The four species typically grow at elevations below 2000 m [5]. *P*. *spina-christi* Mill. is exclusively distributed in the meridional and submeridional regions of southern Europe and western Asia [1] at elevations below 2300 m [4]. *P*. *spina-christi* is also cultivated as an ornamental plant or hedge in Qingdao (Shandong Province), China [5] and North Africa [4]. *Paliurus* has broad ecological tolerance in China and occurs mainly in the southwest, central-south and east regions, with a northernmost extension in Gansu Province [5].

The fossil record for *Paliurus* is extensive in the Cenozoic of the Northern Hemisphere, including western North America, central Europe and eastern Asia, and its age ranges from the Eocene to Pliocene [4]. Asia includes a long fossil history of *Paliurus* since the Middle Eocene, which is based on leaves and fruits from Ube flora, Japan [6] and based on leaves from Liaoning, China [7]. Burge and Manchester [4] emended the diagnosis of *P*. *favonii*, which was recognized by Unger [8] based on morphological analyses, and it was proposed that all Asia fruit records within the *P*. *favonii* morphological variation range were included in this species. *Paliurus* has been broadly distributed geographically since the Eocene from North America and Asia, became widespread and morphologically diverse through the Miocene. *Paliurus* fruit fossils are rarely encountered in China, particularly in the Oligocene sediments. Fruit fossils can provide reliable data for deducing the evolutionary and biogeographical history of a genus, even a family.

Rhamnaceae is a cosmopolitan family that tends to be observed in xeric regions and may be classified as mesothermal [9]. *Paliurus* fossils have been frequently observed in mesic ecological settings from tropical to warm temperate regions [4], which is consistent with the ecological requirements of the extant species. In Asia, the geographical distribution of *Paliurus* macrofossils indicates that the genus has grown in a warm temperate climate since the mid-Eocene [10]. The winged fruits of *Paliurus* are well-adapted for wind dispersal and are important to the reproductive ecology and early diversification of the genus.

In this paper, we identify and describe a new occurrence of *Paliurus favonii* Unger from the Oligocene Ningming Formation of Ningming Basin, Guangxi Province, South China based on the distinctive fruit features; this is the lowest latitude distribution for the species. Our finding provides additional fossil evidence for understanding the evolution of *Paliurus*. Moreover, the phytogeographic history of *Paliurus* is discussed.

## Materials and Methods

### Geological setting

Three *Paliurus* fruit fossils were collected from the shallow lacustrine deposits of the Ningming Formation in the Ningming Basin (at 22°09′15.77″N, 107°01′23.11″E), Guangxi Zhuang Autonomous Region, South China (Fig 2). The Ningming Formation is composed primarily of gray-dark gray mudstone with light yellow shaly siltstone and fine-grained sandstone. The precise age remains unknown due to a lack of volcanic rocks and mammals in the Ningming Formation stratum. Presently, scholars consider two hypotheses for the age of the Ningming Formation. On the one hand, the Ningming Formation is considered to be Miocene based on fish and bivalve fossils [11]; on the other hand, it is also considered to be of the Late Eocene to Oligocene based on the palynological assemblages [12]. Although the Ningming Formation age is hotly debated, previous studies on plant and fish fossils from the same stratum (e.g., *Palaeocarya* [13, 14], *Bauhinia* [15, 16], *Cephalotaxus* [17], *Cupressus* [18], *Calocedrus* [19], *Ailanthus* [20], *Laurophyllum* [21], *Buxus* [22], *Chuniophoenix*, *Livistona* [23], *Ecocarpia* [24] and *Huashancyprinus* [25]) support the notion that the Ningming Formation belongs to the Oligocene age, which is adopted in this paper.

## Material and preparation

These specimens were preserved as compression fossils. Photographs were collected using a Sony DSC-T70 digital camera and examined in detail under a Leica MZ 12.5 stereomicroscope. The extant specimens for morphological comparison are from the herbarium of the School of Life Sciences, Lanzhou University and the South China Institute of Botany, Chinese Academy of Sciences. All specimens are preserved at the Laboratory of Paleontology in Lanzhou University.

The fossil specimen cuticles were prepared by removing residual mineral matrix with 30% HCl and HF, successively, and bleaching with 65% HNO_3_ followed by treating with 5% KOH to obtain the cuticle. After washing several times, a portion of the cuticle samples was dyed and then mounted on thin slides for observation by light microscopy. The other cuticles were dehydrated and mounted on stubs for examination by SEM. The extant specimens’ cuticles were prepared using a 1:1 solution of glacial acetic acid and 30% H_2_O_2_. The prepared cuticles for both the fossil and extant specimens were observed and photographed using a Leica DM4000B multifunctional biological microscope.

The maps of the modern distribution and fossil locality were drawn using CorelDraw X6 software. The palaeogeographic map that shows the *Paliurus* macrofossil distribution was plotted using ODSN software. The fruit morphology terms are from Harris and Harris [26] and Burge and Manchester [4]; Conover [27] was used for the fruit cuticle term. The fruit dimensions were calculated in accordance with Burge and Manchester [4]: A fruit diameter, the average distance across the fruit wing based on two perpendicular measurements; B wing width, the average width of the wing measured at four points 90^°^; and C receptacle diameter, the average distance across the receptacle based on two perpendicular measurements.

### Ethics statement

All necessary permits were obtained for the described sampling sites in verbal or written form. Three *Paliurus* fossils were collected in Ningming County, Guangxi Province, South China. The field work was permitted by the local government. For the extant plant sampling sites, permits were verbally obtained from the herbarium office of the School of Life Sciences, Lanzhou University, China and the South China Institute of Botany, Chinese Academy of Sciences. The extant plant materials did not involve endangered or protected species.

## Results

### Family

Rhamnaceae Jussieu

### Tribe

Paliureae Reissek ex Endl.

### Genus

*Paliurus* Miller

### Species

*Paliurus favonii* Unger

**Fig 3:**
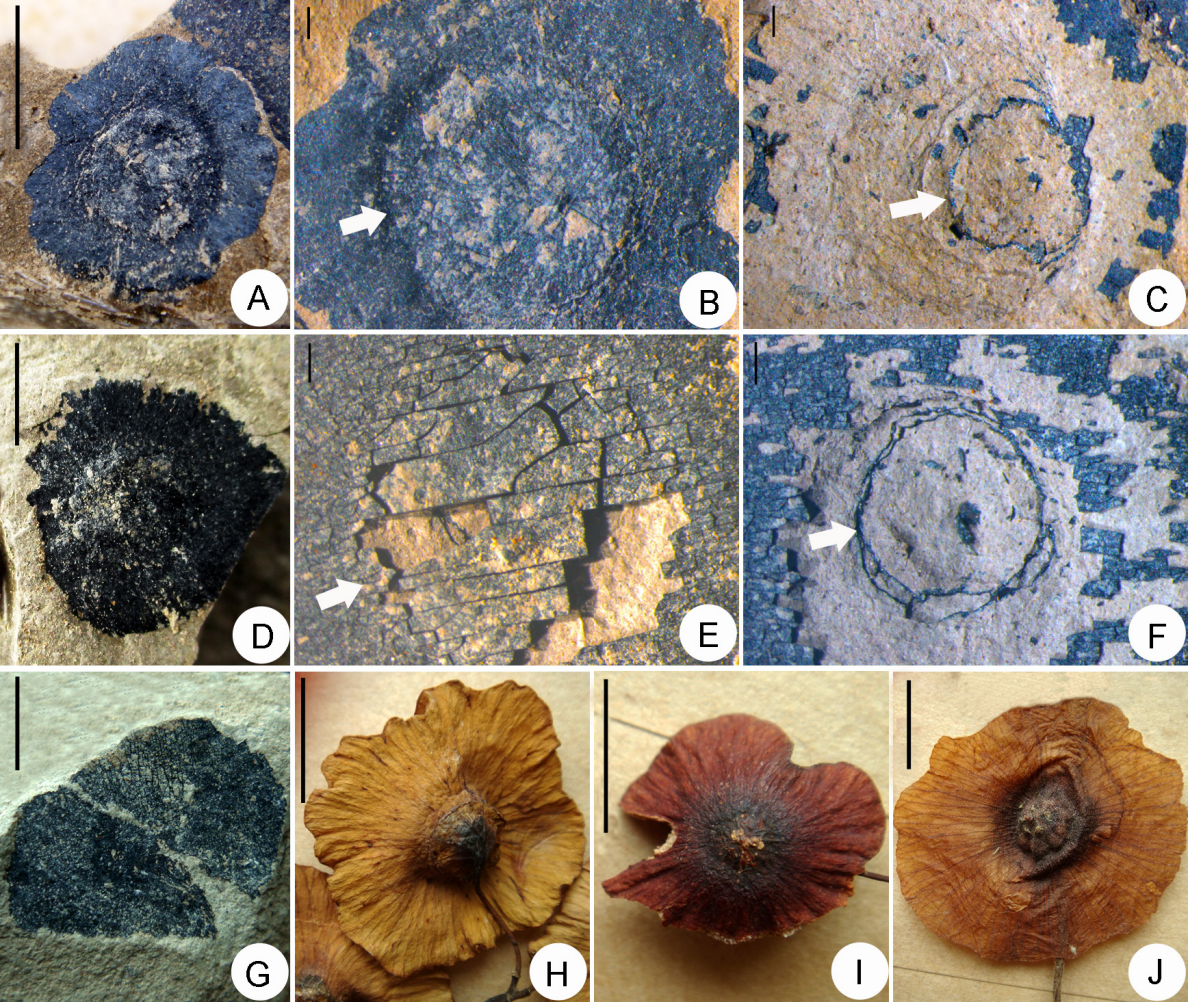
Morphological characteristics of *Paliurus favonii* and similar extant species for morphological comparison. A–G. Morphological characteristics of *Paliurus favonii*. A. Specimen no. LDGSW-2014-222. D. Specimen no. LDGSW-2014-223. G. Specimen no. LDGSW-2014-224. B, E. Showing the distinctly domed and indehiscent endocarp (arrow). C, F. Showing obvious persistent receptacular rim (arrow). H. Extant *Paliurus hemsleyanus*. I. Extant *Paliurus orientalis*. J. Extant *Cyclocarya paliurus* (Juglandaceae). A, D, G. Scale bars = 5.0 mm. B, C, E, F. Scale bars = 1.0 mm. H–J. Scale bars = 10.0 mm.

**Fig 4:**
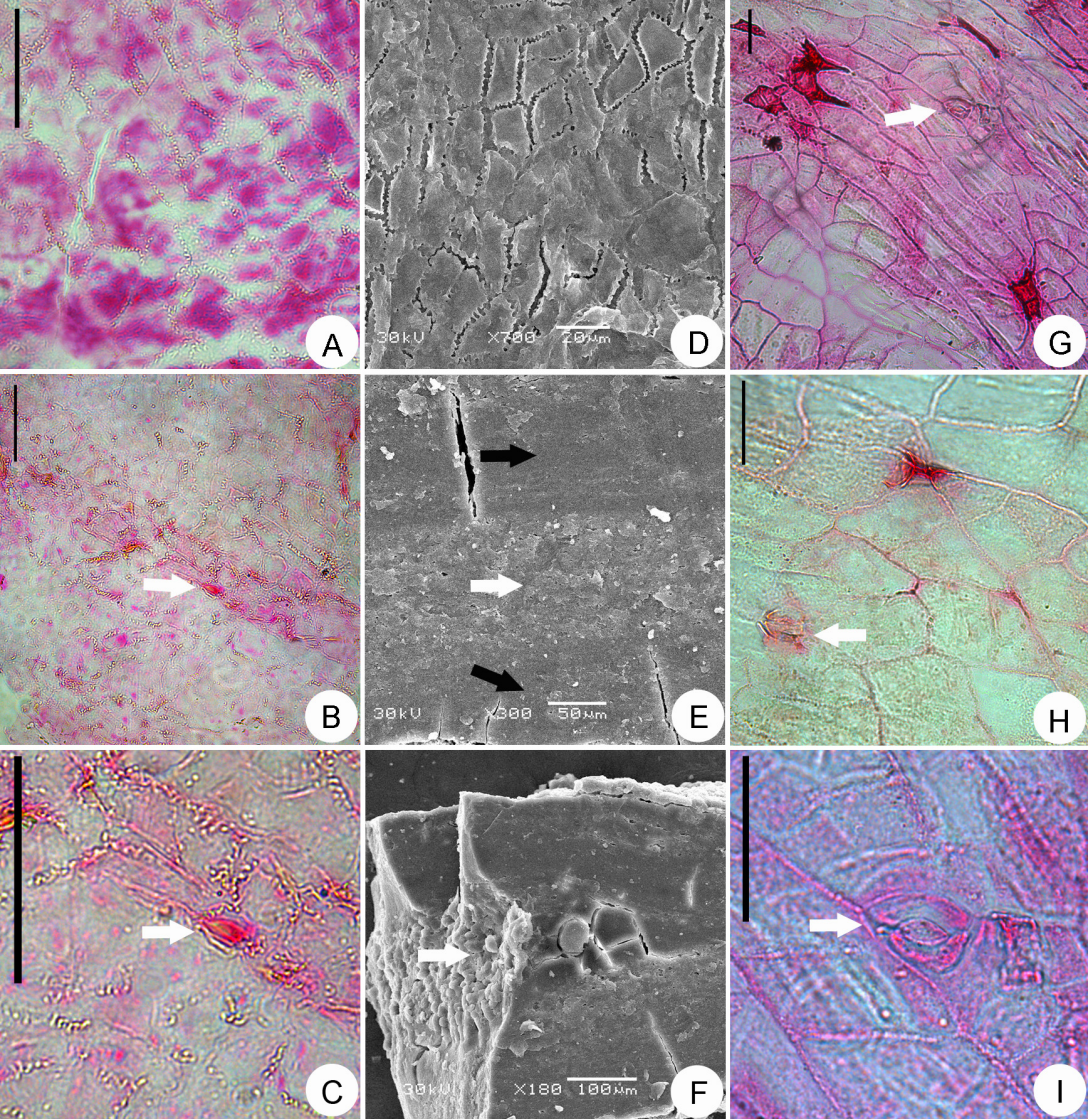
Anatomical characteristics of *Paliurus favonii* and *Paliurus hemsleyanus*. A–C. Endocarp epidermal characteristics of *Paliurus favonii* under light micrographs, showing undulate cell walls. C. Magnification of stomatal apparatus (arrow) from B (arrow), showing anomocytic stoma. D. Epidermal characteristics of *Paliurus favonii* under SEM. E, F. Internal anatomy of *Paliurus favonii* under SEM, showing three layers structure of the endocarp. G–I. Wing epidermal characteristics of extant *Paliurus hemsleyanus*, showing anomocytic stoma (arrow). A–C, G–I. Scale bars = 50.0 μm.

**Figs** 3A–3G; 4A–4F;

### Specimen No

LDGSW-2014-222, LDGSW-2014-223, LDGSW-2014-224.

### Lectotype

LMJ (Landesmuseum Joanneum, Graz) 76518, designated by Kovar-Eder et al. [28], pl. 11, fig.7. Same specimen figured by Unger [8], p. 147, pl. 50, fig. 6.

### Age

Oligocene

### Number of specimens studied

Three

## Description

The fruit is a winged drupe in which the indehiscent endocarp is encircled by the orbicular wing with an irregular undulate margin and 10.0–11.5 mm in diameter. The endocarp in the apical view is distinctly domed, 5.0–6.0 mm in diameter and 1.0 mm deep. The pedicel is not preserved; only a round attachment scar remains on the base of the ovary (Fig 3C and 3F). The receptacle is flat with a pronounced rim surrounding a round pedicel scar; the receptacle is 3.0–4.0 mm in diameter. The vascular ridges radiating from base of the pedicel to the edge of the receptacle form a persistent scar on the basal side of the ovary that can be observed but is not inconspicuous in fossils (Fig 3C). The wing is typically thin with fine radiating, dichotomizing, and anastomosing venation, 2.5–3.0 mm in width, chartaceous, and oriented perpendicular to the main axis of the fruit. The edges of the wing show obvious lobes (Fig 3A). Structurally, the fruit pericarp is composed of three distinctive layers (Fig 4E and 4F). The epicarp is glabrous, and the two-layered mesocarp is thickest and composed of spongy and palisade parenchyma. Due to the lignified endocarp, the inner structure was not preserved.

The epidermal cells of the endocarp are quadrilateral with oblique end walls, have an irregular shape, are somewhat elongate and are typically arranged in longitudinal files. The size is more or less uniform, 11.0–40.0 μm long and 3.0–18.0 μm wide with a length/width ratio up to 9.0 (Fig 4A–4D). The periclinal walls are even, have wax ornamentations on the surface, are mostly rectangular and are slightly elongate or polygonal. The anticlinal walls appear distinctly deep-sinuous with a U shape (Fig 4D) and are thickened. The stomata are sparse, elliptic, and approximately 13.0 μm long and 8.5 μm wide. The stomata are anomocytic, and the guard cells are slightly sunken (Fig 4C).

### Affinities

#### Affinities with extant taxa

The morphological characteristics of *Paliurus* fruit include a circular or orbicular wing encircling a central endocarp with a superficial similarity to *Cyclocarya* Iljinskaya (Juglandaceae), *Asteranthos* Desfontaines (Lecythidaceae) and *Dioncophyllum* Baillon (Dioncophyllaceae) [4, 10, 29, 30]. However, the *Paliurus* fruit can be distinguished by its bi- or tri-locular endocarp and persistent receptacular rim or its persistent scar around the base of the ovary [4, 30, 31]. However, for *Cyclocarya paliurus* (Batal.) Iljinsk., the receptacular rim is lacking, and a single locule is partitioned at its base into 2 or 4 compartments [30, 31]. The wing venation is mainly subparallel (radiating), occasionally anastomoses and does not dichotomize before the medial part [30, 32]. *Asteranthos* Desf. is a Neotropical genus and contains a single species, *A*. *brasiliensis*, which is a species native to Amazonian Brazil and Venezuela [33]. Its fruit is globose to ovate, monospermous and indehiscent; the wing is membranaceous, and the calyx is persistent on the fruit as a circular rim [10, 34, 35]. *Dioncophyllum thollonii* Baill. is a member of the family Dioncophyllaceae, which is predominantly restricted to western Africa tropical rainforests. Its ovary is unilocular [36], and the fruit shape is disk-shaped with a larger ellipsoidal wing that is approximately 5.0–12.0 cm in diameter (Table 1; [10, 37]).

**Table 1:** Comparision between *Paliurus* and other taxa with similar fruit morphology.

Characteristics	*Paliurus*	*Dioncophyllum*	*Cyclocarya*	*Asteranthos*
shape of fruit	disk-shaped	disk-shaped	disk-shaped	globose to ovate
wing texture	chartaceous to leathery	leathery	leathery	membranaceous
number of locules	2–3	1	1 or 2	1
vascular bundles of hypanthium	present	present	absent	present
venation of wing	subparallel (radiating)	subparallel (radiating)	subparallel (radiating) anastomosing	subparallel (radiating)
shape of wing	circular to ellipsoidal	broadly circular to ellipsoidal	circular to ellipsoidal	circular to ellipsoidal
receptacle rim	persistent	*–*	absent	persistent
pericarp structure	3-layer	3 or 4 -layer	3-layer	3-layer
dispersal mode	wind dispersal	wind dispersal	wind or water dispersal	water dispersal

Five extant *Paliurus* species can be partitioned into two groups based on their fruit morphology and dimensions. The first group is the *P*. *spina-christi* group, which is comprised of *P*. *spina-christi*, *P*. *orientalis* and *P*. *hemsleyanus*, and the second group is the *P*. *ramosissimus* group, which consists of *P*. *ramosissimus* and *P*. *hirsutes* [4, 5]. In the *P*. *spina-christi* group, the wing is thin, chartaceous, 2.0–10.0 mm in width, and equatorially extended with limited development of spongy parenchyma in the mesocarp [4]. The fruit dimensions are much larger than the fossil specimens (Table 2). However, the wing index is 0.2–0.3, which is similar to our specimens. In the *P*. *ramosissimus* group, the wing is thick, robust, and wedge-shaped at the longitudinal section with a well-developed spongy parenchyma in the mesocarp, and the fruit is pubescent. By comparing the fossil specimens with the two extant groups, the present specimens are more similar to the first group based on the wing texture, fruit morphology and dimensions, and wing/fruit and receptacle/fruit indexes.

To more precisely assign the present fossils, we compared our specimens with the cuticular characteristics of the extant species. The fossil species are almost identical to the extant species in orientation and stomata type (Fig 4C and 4I), but they differ in the epidermal cell wall morphology. The anticlinal walls of the present specimens are distinctly deep-sinuous (Fig 4D) and thickened, which differ from the extant species that feature almost straight anticlinal walls (Fig 4G–4I).

**Table 2:** Morphological characteristics of extant and fossil *Paliurus* species (characteristic data from Burge and Manchester [4]).

Species	Fruit diameter (mm)	Mean	Wing	Wing wide (mm)	Wing index	Receptacleindex	Fruit pubescence
*P*. *hirsutus*	9.0–17.0	14.7	thick	1.5–4.0	0.2	0.3	yes
*P*. *ramosissimus*	11.0–18.0	14.0	thick	1.0–2.5	0.1	0.3–0.4	yes
*P*. *orientalis*	11.0–20.0	14.2	thin	2.0–8.0	0.2–0.3	0.2–0.3	no
*P*. *hemsleyanus*	19.0–35.0	25.4	thin	7.0–10.0	0.3–0.4	0.2	no
*P*. *spina-christi*	15.0–35.0	21.5	thin	4.0–7.0	0.2–0.3	0.2–0.4	no
Present specimens	10.0–11.5	10.5	thin	2.5–3.0	0.2–0.3	0.3–0.4	no

#### Affinities with fossil taxa

*Paliurus* fossil fruits were reported as abundant in the Cenozoic deposits of the Northern Hemisphere and presented varied morphologies and dimensions. Burge and Manchester [4] made a detailed summary and emended the diagnosis of *P*. *favonii*. They concluded that, except for *P*. *clarnensis*, most *Paliurus* fruits including European, Asian, and North American records ranging from the Eocene to Pleistocene, should be considered the *P*. *favonii* that was described by Unger [8] based on both the fruits and leaves from the Miocene deposits near Parschlug, Austria. All Asian fruit dimensions and wing widths fall within the *P*. *favonii* morphological continuum. The morphological analysis data (e.g., wing width, fruit diameter, receptacle diameter, wing index, receptacle index, and endocarp depth) of the present fossil fruits fall within the *P*. *favonii* morphological continuum (Table 3), thus the present specimens should be considered *P*. *favonii* species. *P*. *favonii* spans and even exceeds the entire range of modern species [4].

**Table 3:** Morphological characteristics of present and other known fossil specimens (characteristic data from Burge and Manchester [4], Li et al. [10] and Correa et al. [30]).

Species	Fruit diameter (mm)	Endocarp(mm)	Receptacle diameter (mm)	Wing width (mm)	Wing index	Receptacle index	Endocarp depth (mm)	Vascular bundles of hypanthium	Shape of endocarp	Margin of wing	Venation of wing
*P*. *favonii*	5.7–20.0	2.4–13.5	1.9–5.7	1.1–5.5	0.1–0.3	0.2–0.5	1.0	8–10	hemispheric to lensoidal	–	Subparallel (radiating)
*P*. *clarnensis*	18.5–23.5	6.0–7.3	1.2–1.8	6.3–8.0	0.3–0.4	0.1–0.2	1.0–1.6	8–10	hemispheric to lensoidal	irregular	Subparallel (radiating) fine
*P*. *microcarpa*	8.8 or 10.0	6.3 or 6.5	3.3	1.2 or 1.8	0.1–0.2	0.3–0.4	3.0–4.0	–	hemispheric to lensoidal	undulate	Subparallel (radiating)
*Archaeopaliurus boyacensis*	35.0	12.0	–	11.5	0.3	–	4.0	8–10	hemispheric to lensoidal	entire	subparallel (radiating) anastomosing
Present specimens	10.0–11.5	5.0–6.0	3.0–4.0	2.5–3.0	0.2–0.3	0.3–0.4	1.0	–	hemispheric to lensoidal	undulate	subparallel (radiating)

The present specimens are similar to *Paliurus clarnensis*, *P*. *microcarpa* and *Archaeopaliurus boyacensis* in morphological characteristics. However, certain additional characteristics are distinctive [4, 10, 30]. The *Archaeopaliurus boyacensis* was found in the late Cretaceous (Maastrichtian) of Colombia, northern South America [30]. The endocarp is 12.0 mm in width and 4.0 mm in depth; the fruit is 35.0 mm in diameter. The fruit dimensions are larger than those of the present specimens. *P*. *clarnensis* was reported from the Middle Eocene of Red Gap, Jefferson County, Oregon and overlaps with *P*. *favonii* in fruit and endocarp dimensions [4]. However, the receptacle index of *P*. *clarnensis* is smaller than the present specimens. *P*. *clarnensis* has a larger fruit (i.e., 18.5–23.5 mm) with a narrower receptacle rim (i.e., 1.2–1.8 mm). *P*. *microcarpa* from the Miocene of Tiantai County in Zhejiang Province of China was documented by Li et al. [10] and exhibits clear differences in wing index and endocarp depth (Table 3). The *P*. *microcarpa* endocarp is 3.0–4.0 mm in depth, and the wing index is 0.1–0.2 mm, which is outside the range of the present specimens. Table 3 includes an outline of the fruit characteristics that differentiate the fossil representatives of *Paliurus*.

## Discussion

**Fig 5:**
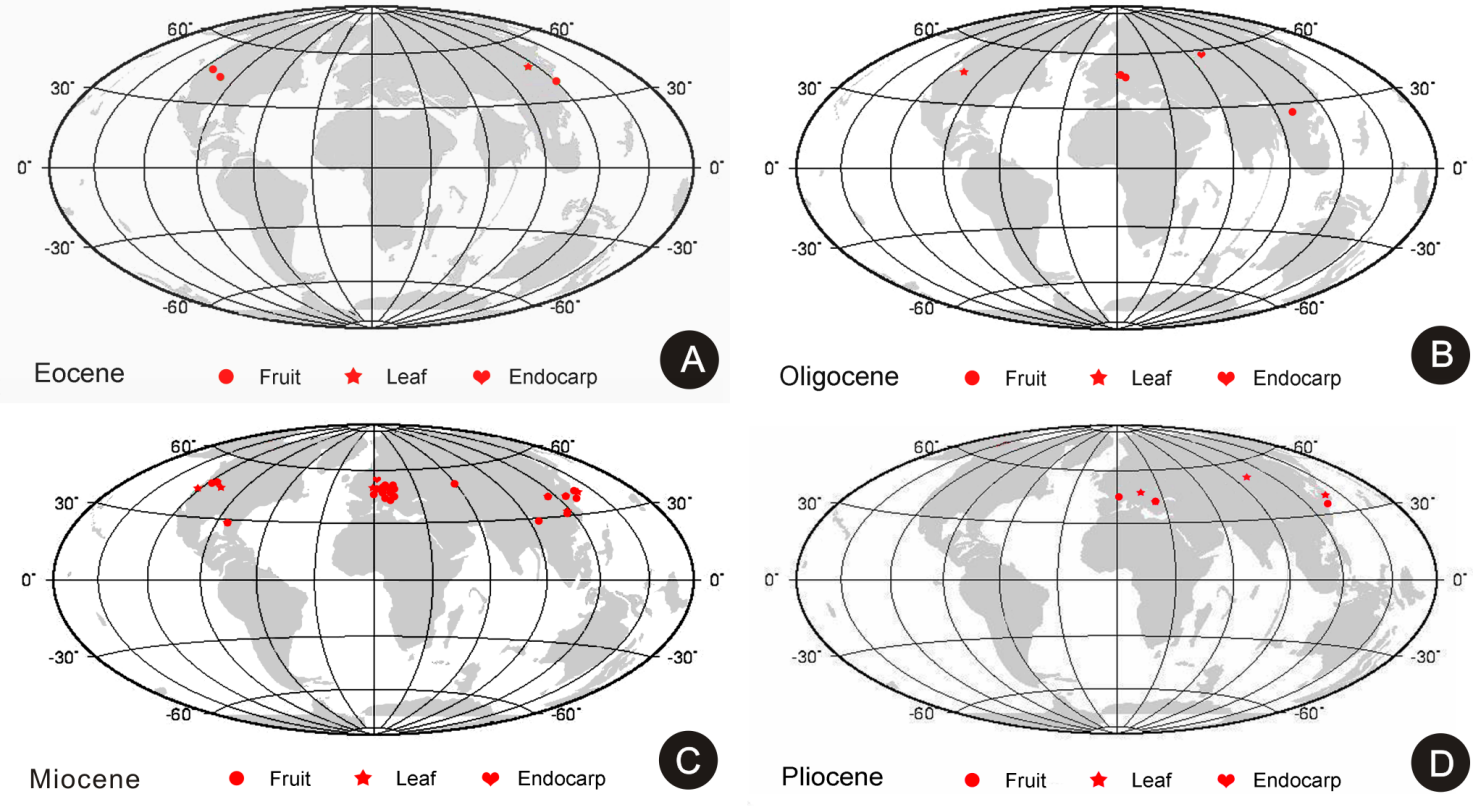
Palaeogeographic distribution of *Paliurus* macrofossils (including fruits, leaves and endocarps) [4, 10, 41]. A. Eocene, at 41.2 Ma. B. Oligocene, at 28.1 Ma. C. Miocene, at 13.82 Ma. D. Pliocene, at 3.6 Ma.

Currently, *Paliurus* is widely distributed in Asia and Eastern Europe [1], but abundant fossil data, including fruits, endocarps, and leaves, indicate a broader distribution in the Cenozoic (Fig 5). The earliest reliable *Paliurus* fruit fossil evidence is from the Early Eocene Wind River Formation of Wyoming, North America [31], and most recent occurrences are from the Pleistocene of Yamashiro Province, Japan [38]. In North America, the *Paliurus* fruits and leaves are from the western and southeastern United States, and the age spans from the Early Eocene to Middle Miocene [31, 39]. In Europe, the *Paliurus* fruits and leaves have been reported from the Czech Republic, Austria, Germany, France, and Poland, and the age spans from the Oligocene to Pliocene [8, 28, 40]. In Asia, *Paliurus* fossil records are from Japan, China, Siberia, and Kazakhstan from the Middle Eocene to Pleistocene [6, 7, 10, 38]. The fossils are well-represented in the Miocene localities, but the Pliocene and Pleistocene records were limited to Europe and Asia [4]. In addition, they were widely distributed in the middle latitudes of the Northern Hemisphere during a large portion of its history.

In China, *Paliurus* fossil records are rare compared with fossil records elsewhere in the world. The earliest report of *Paliurus* was published based on leaves from the Middle Eocene of Fushun, Liaoning [7, 42], and other fossil records including leaves or fruits, were reported from the Early Miocene of Yunnan and Shandong [42], and from the late Middle Miocene to early Late Miocene of Tiantai, Zhejiang [10]. The fossil records are undoubtedly incomplete in China, and, previously, such records from the Oligocene strata of this country did not exist. Our finding reveals the lowest latitude distribution of *Paliurus favonii* and provides a new fossil record, which is the first fossil described with detailed cuticular characteristics in the Oligocene sediment from south China. This recognition of *Paliurus* demonstrates that this genus existed in the Ningming region, South China, where it is currently distributed–by the Oligocene. It further aids in understanding the radiation process of the genus.

Winged fruits that represent various angiosperm families are frequently found in many Cenozoic fossil localities [43, 44]. Fruits that fall from the mother plants may be dispersed by wind with a potential dispersal distance that depends on the tree size, but the dispersal distance rarely exceeds one hundred to a few hundred meters [45, 46]. The *Paliurus* fruit, with a single surrounding wing, facilitates wind dispersal by gliding and undulating but not with significant cumulative forward motion [47]. Many fossil winged fruits that are functionally similar to the winged drupe of *Paliurus* have been reported from low latitude, moist forests in the Ningming Basin, such as *Palaeocarya* (Juglandaceae) [13, 14] and *Ailanthus* (Simarubaceae) [20], which might be associated with wind dispersal. Further, other wind diaspores, such as scattered winged seeds of Pinaceae, *Chaneya* (Rutaceae), *Burretiodendron* (Tiliaceae), *Acer* (Anacardiaceous) *Ulmus* (Ulmaceae) and *Fraxinus* (Oleaceae), have also been found from the Ningming Flora. Furthermore, genera with winged fossils may compose a certain proportion of the pollen sum from the Ningming microflora [12], e.g., *Fraxinoipollenites* 0–2.0%; *Platycaryapollenites* 0–1.9%; *Momipites*, 0.4–2.8%; and *Ulmipollenites*, 1.2–4.6%. The presence of wind-dispersed species suggests that sufficient winds were present in Ningming flora during the Oligocene.
